# Secondary metabolites changes in germinated barley and its relationship to anti-wrinkle activity

**DOI:** 10.1038/s41598-020-80322-0

**Published:** 2021-01-12

**Authors:** Sang Cheol Park, Qianwen Wu, Eun-yi Ko, Ji Hwoon Baek, Jeoungjin Ryu, Seunghyun Kang, Mi Kyung Sung, Ah-Reum Cho, Young Pyo Jang

**Affiliations:** 1 Department of Life and Nanopharmaceutical Sciences, Kyung Hee University, Seoul, 02447 Republic of Korea; 2 Dermapro Bio Research Center, DERMAPRO Ltd., 213-3 Chumdan-ro, Jeju-si, Jeju-do 63309 Republic of Korea; 3 Dermapro Skin Research Center, DERMAPRO Ltd., 30 Bangbaejoongang-ro, Seocho-gu, Seoul, 06684 Republic of Korea; 4 Cosmax BTI R&I Center, Bio Material Research Team, Seongnam-si, 13486 Republic of Korea; 5 Durae Corporation, Durae R&D Center, Gunpo-si, 15847 Republic of Korea; 6 Durae Corporation, Jeju Bio Center, Jeju-si, 63359 Republic of Korea; 7 Department of Oriental Pharmaceutical Science, Kyung Hee University, Kyungheedae-ro 26, Dongdaemun-gu, Seoul, 02447 Republic of Korea

**Keywords:** Secondary metabolism, Statistical methods

## Abstract

The purpose of this research was to identify metabolite change during barley (*Hordeum vulgare*) germination and reveal active principles for the anti-wrinkle activity. Barley was germinated with deionized water (DW) and mineral-rich water (MRW) for the comparison of the effect of mineral contents on the metabolites changes during germination. The effects of germinated barley extracts (GBEs) on collagen production and collagenase inhibition were evaluated in vitro using human dermal fibroblasts (HDFs). A pronounced anti-wrinkle activity was observed in the test group treated with the MRW-GBEs. In order to find out the active components related to the anti-wrinkle activity, an orthogonal projection to latent structure-discriminant analysis (OPLS-DA) was performed, using the data from secondary metabolites profiling conducted by UPLC–PDA–ESI–MS. The anti-wrinkle activity of MRW-GBEs was revealed to be associated with the increase of oligomeric compounds of procyanidin and prodelphinidin, indicating that it can be used as an active ingredient for anti-wrinkle agents.

## Introduction

Germination refers to the process that seeds emerge from a period of dormancy and begin to sprout in an environment suitable for growing. During the process of germination, various enzymes, new proteins, vitamins, and phytochemicals are produced to supply the necessary nutrients for vital activities of seeds after germination^1^. Metabolome produced in this process have been reported to have biological activities including antioxidant, anti-inflammatory^2^, and anti-aging activity^3^, etc. Thus, germinated seeds, especially eatable germinated grain seeds are receiving high attention as sources for the active ingredients used in healthy functional foods and cosmeceuticals^4^.

Barley (*Hordeum vulgare* L.) is a cereal plant belonging to the grass family (Poaceae). It is the fourth largest grain crop grown in temperate regions worldwide^5^. Barley is considered as one of the most important cereals due to its versatility. It is used as not only human food and animal feed but also as a material to make beer or certain distilled beverages. Recently, increasing interests were drawn to barley for its antioxidant activity derived from the high content of phenolic compounds^6,7^. Besides, it has also been reported that germinated barley possess anti-inflammatory activities^8^.

Water is essential for the germination as well as the growth of the plants. Comparing with deionized water (DW), the mineral-rich water (MRW) prepared from lava seawater found in Jeju island of South Korea contains not only a large content of essential minerals such as iron, manganese, zinc, calcium, sodium, and potassium but also many useful trace minerals derived from volcanic rocks^9^ such as vanadium, germanium, and selenium. It has been reported that MRW can promote growth of the plant^10^.

Collagen occupies 70–80% of the dermis layer, provides strength and elasticity to the skin that protects the skin from external stimuli or forces. Type I and III collagen are the major members of collagen in the human dermis layer, which is 80% and 15% of total collagen, respectively^11^. In normal skin, the synthesis of type I collagen is in equilibrium with the decomposition of type I collagen by Matrix metalloproteinase-1 (MMP-1) to maintain skin elasticity. Wrinkles are known to be formed when this balance is broken by aging or external factors^12^.

Metabolomics has been applied intensively in the field of natural products related science in order to classify the variables and identify the marker molecules according to their phenotypic characteristics. Principal component analysis (PCA) and Orthogonal projection to latent structures discriminant analysis (OPLS-DA) are two important multivariate data analysis approaches commonly used in metabolomics. Unlike PCA that is working with unprocessed analytical data, the non-correlated variation was removed in OPLS-DA to make model interpretation easier when the identity of sample is known. This characteristic makes OPLS-DA more appropriate to find marker components that explain different biological activities among samples since the dependent variable is selected to represent class^13–15^.

In this study, we intended to explore the possibility of germinated barley extracts (GBEs) as an active ingredient for the cosmeceutical products. We germinated barley with different water source, DW and MRW to maximize the biological efficacy and then investigated the anti-aging activities of the extracts on human dermal fibroblast cells (HDFs). Since the inhibition of MMP-1 secretion and increase of procollagen type I production (PIP) in HDFs is a typical indicator of anti-wrinkle activity, it was evaluated by treating HDFs with the GBEs. At the same time, an orthogonal projection to latent structure-discriminant analysis (OPLS-DA) was performed on the data from secondary metabolites profiling conducted by UPLC–PDA–ESI–MS to find out the active components related to the anti-wrinkle activity.

## Materials and methods

### Chemicals and reagents

Dulbecco’s modified Eagle’s medium/F12 nutrient mixture Ham (DMEM/F12 3:1), antibiotic–antimycotic solution, trypsin–EDTA, phosphate buffered saline, Hank’s Balanced Salt Solution (HBSS) and fetal bovine serum (FBS) were purchased from Gibco (Grand Istand, NY, USA). 3-(4,5-Dimethylthiazol-2-yl)-2,5 Diphenyl-2H-tetrazolium Bromide (MTT), phosphoramidon disodium salt (Phosphoramidon), ( +) sodium l-ascorbate (l-Ascorbate), epigallocatechin gallate (EGCG), *N*-succinyl-tri-alanyl-p-nitroanilide (STANA), albumin from bovine serum (BSA), Bradford reagent and trifluoroacetic acid (HPLC grade) were obtained in Sigma-Aldrich (St. Louis, MO, USA). Besides, dimethyl sulfoxide (DMSO) was purchased from Amresco (Solon, OH, USA). Triton X-100 was obtained from Yakuri Pure Chemicals (Tokyo, Japan). Ethanol (HPLC grade) was obtained from Duksan Chem. Co. (Seoul, South Korea). Acetonitrile (HPLC grade) was purchased from Fisher Scientific Korea (Seoul, South Korea). Procollagen type I peptide (PIP) ELISA kit was obtained from Takara (Tokyo, Japan). Matrix Metalloproteinase-1 (MMP-1) Human ELISA kit was purchased from Abcam (Cambridge, MA, USA). YOKUDELNA calibration kit was obtained from JEOL Ltd. (Tokyo, Japan).

### Sample preparations

Barley (*Hordeum vulgare*) was purchased from JBT Pharmaceutical Manufacturing Co., Ltd (Jeju, South Korea). Mineral-rich water (MRW) with 5000 hardness was provided by Magma Seawater Industrialization Support Center (Jeju, South Korea). In order to use an optimal hardness of MRW to germinate seeds, the MRW with 5000 hardness was diluted to the hardness of 400.

Nine hundred gram of barley soaked in DW for 3 h in the purpose of making the seeds absorb enough water to reach the water content that can germinate normally. The soaked seeds then were divided into nine groups, with 100 g for each group. One group was used as the blank control group (B1) which is not germinated. Eight groups were germinated for 24 h, 48 h, 72 h and, 96 h by DW (B2-B5) and MRW (B6-B9), respectively. There was no observational change of seeds before 24-h germination. The phenotypic changes of germinated seeds according to the germination period were photographed and the length of root was measured and supplied as a supplementary file (Supplementary Figure S1 and Supplementary Table S2). After that, all of the seeds were washed, dried, ground into powder and then extracted with 1 L of 70% ethanol for 6 h by stirring, respectively. All the extracts were filtered and evaporated to powder by using a rotary evaporator to prepare the DW germinated barley extract (DW-GBE) and MRW germinated barley extract (MRW-GBE). The powder of extracts was stored at 20 °C before use.

### Cell culture

Human Dermal Fibroblast cells (HDFs) were obtained from American Type Culture Collection (Manassas, VA, USA). HDFs are primary cells isolated from adult skin without the detection of Mycoplasma, Hepatitis B, Hepatitis C, HIV-1, Bacteria, yeast and other fungi. The HDFs were cultured in DMEM / F12 3: 1 mixed medium with 1% Antibiotic–Antimycotic (v/v) and 10% FBS (v/v) at 37℃ in an incubator (HERAcell 150i, Thermo Scientific, Waltham, MA, USA) containing 5% CO_2_.

### Cytotoxicity of GBEs on human dermal fibroblast cells (HDFs)

Nine samples (B1 to B9) were diluted to the concentrations of 0, 10, 20, 40 μg/mL for subsequent experiments. Cell viability was evaluated using MTT assay. HDFs were cultured in 24-well plates at a density of 5 × 104 cells/well and incubated in complete culture medium for 24 h. After that, cells were treated with different dose of the samples for another 6 h and then incubated with 100 µL of 0.5% MTT solution for 4 h. The culture media was removed and 500 μL of DMSO was added, shaking for 10 min. The absorbance was measured at 570 nm in ELISA reader (Sunrise, Tecan, Grödig, Austria).

### Quantification of total protein content

The total protein quantification of HDFs was conducted by Bradford method using BSA as the standard solution. BSA was diluted stepwise to 0, 2, 5, 10, and 20 µg/mL in distilled water and 20 µL of each concentration of BSA solution was added to 0.98 mL of Bradford reagent. After reacting for 5 min, absorbance was measured at 595 nm and BSA standard curve was drawn. The absorbance of the cell lysate of HDFs which were cultured in 24-well plate was measured in the same manner. The total protein contents in HDFs were calculated using the BSA standard curve.

### Assay of collagen type I synthesis

For PIP measurement, HDFs were cultured in 24-well plates at a density of 5 × 104 cells/well and incubated in complete culture medium for 24 h. Then, cells were treated with various concentrations of each sample (1, 5, 10 µg/mL) for another 24 h. After that, the supernatant was taken and the content of procollagen was measured at 450 nm by using PIP ELISA Kit. PIP contents were calibrated by total protein content and compared with l-ascorbate (30 µg/mL) as the positive control.

### Assay of MMP-1 secretion

Human dermal fibroblast cells (HDFs) were cultured in 24-well plates at a density of 5 × 104 cells/well and incubated in complete culture medium for 24 h. Then, cells were treated with various concentrations of each sample (1, 5, 10 µg/mL) for another 48 h. After that, the supernatant was taken and the inhibition activity of collagenase was measured at 450 nm by using MMP-1 Human ELISA Kit. The contents of MMP-1 were calibrated by total protein content and compared with EGCG (2.3 µg/mL) as the positive control.

### Statistical analysis

Statistical analysis was performed using the SPSS Package Program (Version 25, IBM, Chicago, IL, USA). All of the values were obtained from at least three independent studies and expressed as means ± standard deviation (SD). Statistical analysis was determined using independent samples T-test in the statistical packages. Statistical significance was set at *p < 0.05. Prism Software 4 (Graphpad Software, San Diego, CA) was used for graphing.

### UPLC–PDA–ESI–TOF–MS analysis

Fifty milligram of each sample was dissolved in 1 mL 70% ethanol and sonicated for 60 min. Then, the solution was filtered with 0.2 µm polytetrafluoroethylene filter (Whatman International Ltd, Maidstone, UK) to get the stock solution to inject into the UPLC system.

Analysis of the stock solution was carried out by UPLC system (Acquity H-class, Waters Corp., Milford, MA, USA) using an analytical column (Acquity UPLC HSS T3 column, 1.8 µm, 2.1 × 50 mm, Waters Corp., USA). The photodiode array (PDA) detector was recorded between 210 and 400 nm. The monitoring wavelength was 330 nm. The mobile phase was comprised of Acetonitrile (solvent A) and distilled water with 0.1% trifluoroacetic acid (solvent B). The gradient condition was 0–2 min, 6%; 2–7 min, 6% to 10%; 7–15 min, 10% to 12%; 15–30 min, 12% to 14%; 30–55 min, 14% to 35%; 55–65 min, 35% to 100% as percentage of solvent A. The column temperature was 25℃. The flow rate was 0.4 mL/min and the injection volume was 2.0 µL.

Molecular weight and molecular formula of differential compounds were measured by TOF–MS (JMS-T100TD, JEOL Ltd., Tokyo, Japan) connected with ESI source (JEOL Ltd., Tokyo, Japan), operated with Mass Center software (version 1.3.7). In the positive mode, the conditions of MS analysis were set as follows: orifice 1 = 80 V, orifice 1 temperature = 80℃; ring lens = 5 V; orifice 2 = 10 V; needle electrode = 2000 V; desolvating chamber temperature = 250℃; detector voltage = 2200 V, peak voltage = 1500 V; the flow rate of nitrogen gas used for nebulizing and desolvating was set to 1 and 3 L/min, respectively. Mass scale calibration was accomplished with the Yokudelna calibration kit for an accurate mass measurements and calculations of the elemental composition, MS acquisition was set with a scan range of m/z 50–1500.

### OPLS-DA analysis

The raw data were exported to an ASCII file using Empower 3 (Waters Corp.) and then aligned using Excel (Microsoft Corp, Redmond, WA, USA). All the Y values (peak area) with the same X value (retention time, keep one decimal place) were added together and represented as a single value to reduce the number of variants. The processed data were exported into the SIMCA-P + (Version 14.1, Umetrics, Umea, Sweden) for orthogonal partial least squares discriminant analysis (OPLS-DA). Pareto scaling methods were performed in this study. Score plot and variable importance in projection value (VIP) were applied to select the potential biomarkers between the two groups.

## Results and discussion

### In vitro anti-wrinkle activity of GBEs on HDFs

Many previous studies have been reported that germinated barley possessed an antioxidant activity derived from the high content of phenolic compounds toward various oxidative stress in vitro and in vivo^16,17^, which has been widely utilized for the development of health foods. To date, other biological activities of germinated barley, especially the anti-wrinkle activity, has not been reported yet. In this study, we have explored the anti-wrinkle activity of GBEs on HDFs by investigating the increment of PIP and reduction of MMP-1.

**Figure 1:**
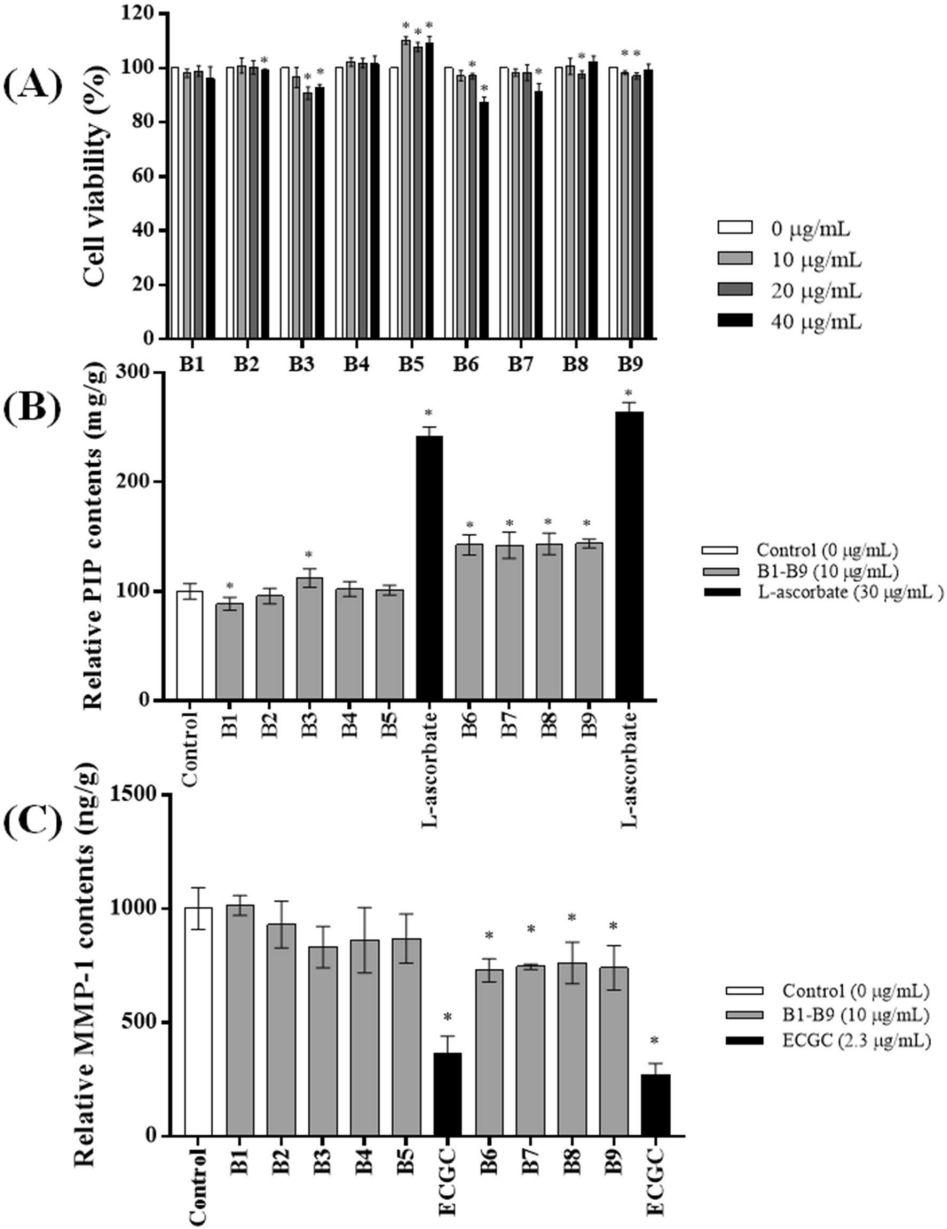
Cell viability and anti-wrinkle activity of GBEs. (**A**) Cell viability of human dermal fibroblasts (HDFs) treated with DW-GBEs (B2–B5) and MRW-GBEs (B6–B9) at different concentration. The data are expressed as % of vehicle control (Mean ± SD, *p < 0.05, Vehicle control vs. Sample extracts). (**B**) Relative PIP contents^1^ in human dermal fibroblasts (HDFs) after treated with different samples at the concentration of 10 µm/mL. The data are expressed as mg/g when vehicle control is set to 100 mg/g (Mean ± SD, *p < 0.05, Vehicle control vs. Sample extracts vs. Positive control^2^). ^1^ Relative PIP contents: the relative PIP contents in HDFs cells after treatment when the relative PIP content of vehicle control is set to 100 mg/g. ^2^ Positive control: l-ascorbate. (**C**) Relative MMP-1 contents^1^ in human dermal fibroblasts (HDFs) treated with GBEs samples at 10 µm/mL. The data are expressed as ng/g when vehicle control is set to 1000 ng/g (Mean ± SD, *p < 0.05, Vehicle control vs. Sample extracts vs. Positive control^2^). ^1^ Relative MMP-1 contents: The relative MMP-1 contents in HDFs cells after treatment when the relative MMP-1 content of vehicle control is set to 1000 ng/g. ^2^ Positive control: epigallocatechin gallate (ECGC). All the charts were drawn by Prism Software 4.

In order to evaluate the cytotoxicity of GBEs in vitro, samples were prepared at various concentrations (10, 20, 40 µg/mL) and used to treat HDFs. All the GBEs showed no significant cytotoxicity compared to that of the control as shown in Fig. 1A. Additionally, cell viability was maintained over 95% when cells were treated with samples at the dose of 10 µg/mL. These findings suggested that samples at the concentration of 10 µg/mL could be used for following in vitro anti-wrinkle activity measurement with no associated cytotoxicity.

The amount of collagen type I synthesis that occurred upon the exposure to GBEs was quantitatively measured by using a procollagen type I peptide (PIP) ELISA kit. As is well known, collagens are synthesized as procollagens. These precursor molecules contain additional peptide sequences at both the amino-terminal end and the carboxy-terminal end, usually called as “propeptides”. These propeptides are cleaved from the collagen triple-helix molecule during its secretion, after which the triple-helix collagens are polymerized into extracellular collagen fibrils. Accordingly, the amount of free propeptide stoichiometrically reflects the amount of collagen molecules synthesized. The experimental group were treated with different samples at 10 µg/mL dose whereas the positive control group were treated with 30 μg/mL dose of L-ascorbate due to its promotional effect on collagen biosynthesis^18^. After the calibration by total protein contents and setting the relative PIP content of blank control to 100 mg/g, the relative PIP contents were obtained and represented in Fig. 1B. The intracellular collagen productions of HDFs treated with MRW germinated group (B6, B7, B8, and B9) were significantly increased, which were 144.44 ± 9.071 mg/g, 142.09 ± 12.040 mg/g, 143.36 ± 9.648 mg/g and 143.73 ± 4.193 mg/g, respectively. The increments are more than 40 mg/g in these four groups.

**Figure 2:**
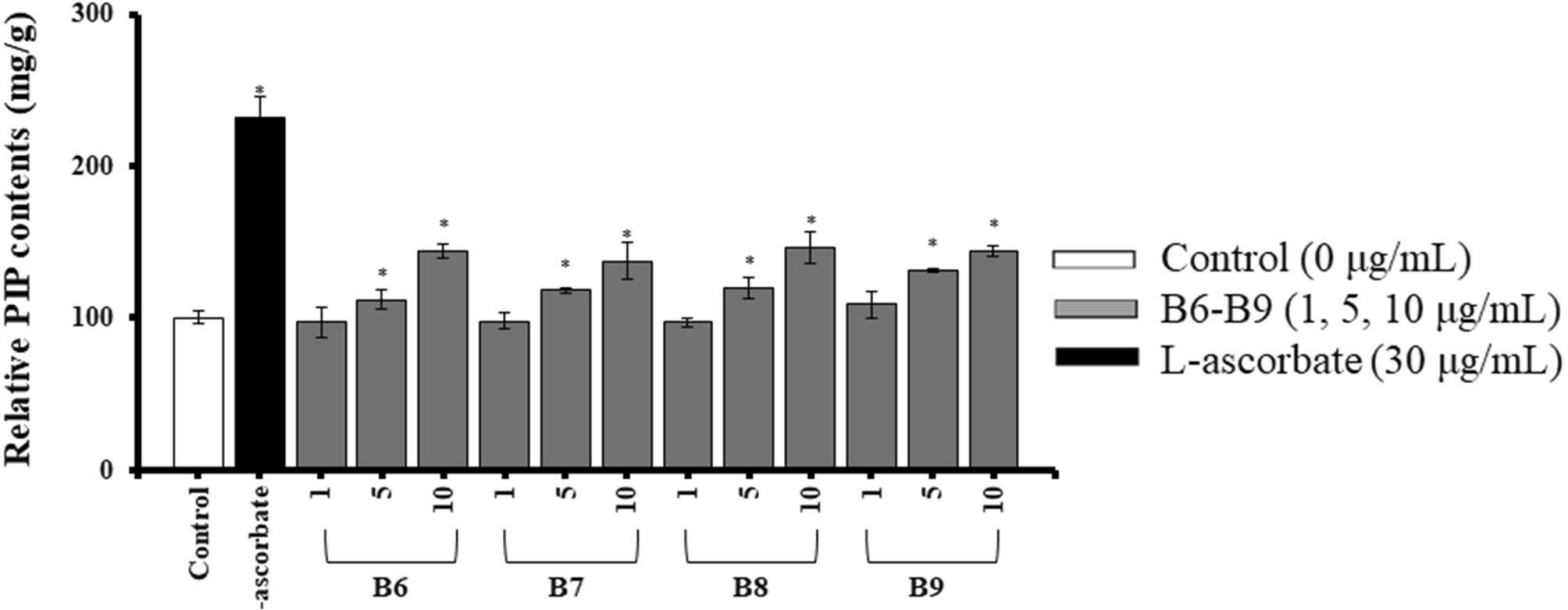
Relative PIP contents^1^ in human dermal fibroblasts (HDFs) after treated with different concentrations of group B6 to B9. The data are expressed as mg/g when vehicle control is set to 100 mg/g (Mean ± SD, *p < 0.05, Vehicle control vs. Sample extracts vs. Positive control^2^). ^1^ Relative PIP contents: The relative PIP contents in HDFs cells after treatment when the relative PIP content of vehicle control is set to 100 mg/g. ^2^ Positive control: l-ascorbate. Chart was drawn by Prism Software 4.

**Figure 3:**
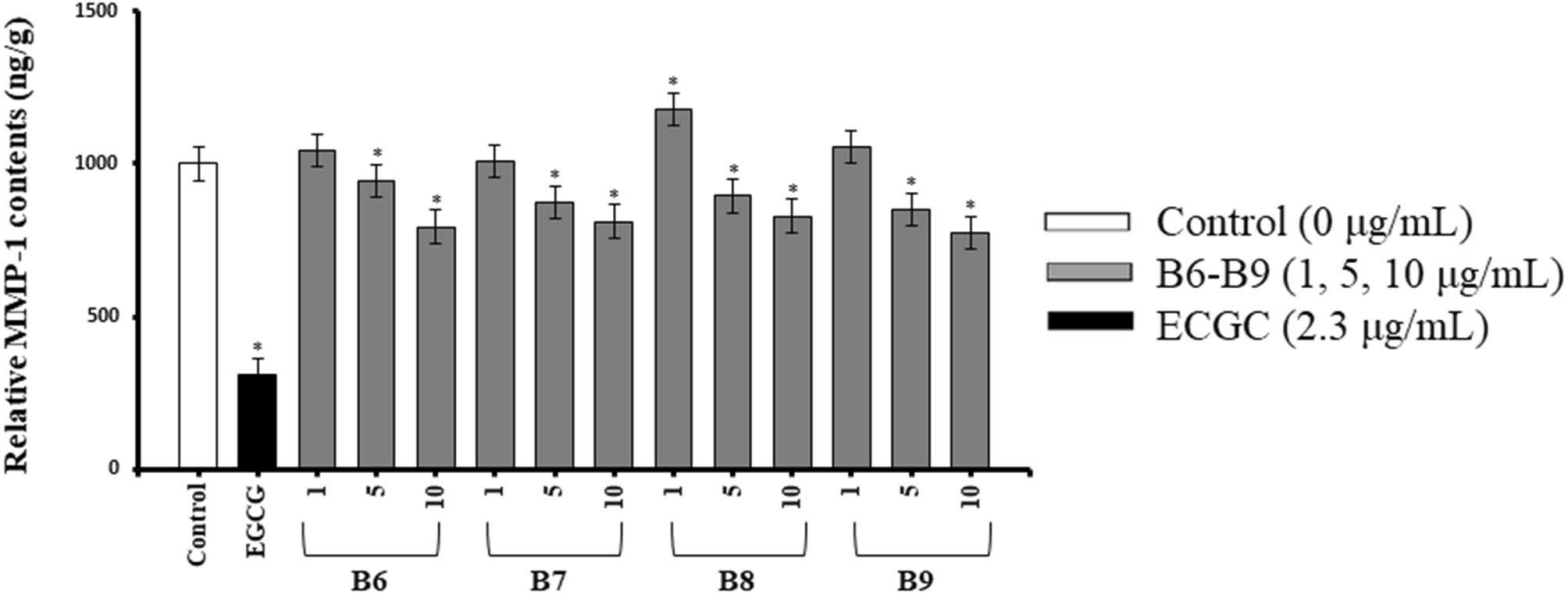
Relative MMP-1 contents^1^ in human dermal fibroblasts (HDFs) treated with various concentrations of group B6 to B9. The data are expressed as ng/g when vehicle control is set to 1000 ng/g (Mean ± SD, *p < 0.05, Vehicle control vs. Sample extracts vs. Positive control^2^). ^1^ Relative MMP-1 contents: the relative MMP-1 contents in HDFs cells after treatment when the relative MMP-1 content of vehicle control is set to 1000 ng/g. ^2^ Positive control: epigallocatechin gallate (ECGC). Chart was drawn by Prism Software 4.

The MMP-1 inhibition activity in HDFs was measured by using Matrix Metalloproteinase-1 (MMP-1) Human ELISA kit. ECGC with 2.3 µg/g dose was used to treat cells as the positive control because it is well known to have an inhibitory effect on collagenase^19^. As shown in Fig. 1C, the contents of MMP-1 were decreased in HDFs treated with B6, B7, B8, and B9. The Relative MMP-1 contents of these four groups were 728.69 ± 50.34 ng/g, 745.23 ± 11.70 ng/g, 761.94 ± 91.37 ng/g and 740.49 ± 98.16 ng/g when setting the blank control to 1000 ng/g. The decrements of MMP-1 contents in these four groups are more than 200 ng/g. In order to see whether these activities were represented by on target manner or not, different concentrations of each samples of B6 to B9 were re-evaluated for same assays. In collagen synthesis assay, all the MRW-GBEs represented dose-dependent promotion of intracellular collagen production which showed on target activity of these group (Fig. 2). From the results of MMP-1 inhibition assay, group B6 to B9 also showed dose-dependent inhibition on MMP-1 contents (Fig. 3). According to the above experiment results, it can be concluded that MRW-GBEs (B6, B7, B8, B9) can efficiently promote the synthesis of collagen type I while inhibiting MMP-1secretion. These findings suggested that MRW-GBEs have a significant anti-wrinkle activity.

### Metabolite changes in GBEs and its relationship to the anti-wrinkle activity

Plenty of previous studies have been conducted on metabolomics profiling of barley (*Hordeum vulgare*), especially about the phenolic compounds mainly by using high-performance liquid chromatography-mass spectrometry (HPLC–MS)^20–22^ or gas chromatography-mass spectrometry (GC–MS)^23,24^. In this study, ultra-performance liquid chromatography (UPLC) analysis coupled with time-of-flight mass spectrometer (TOF–MS) in ESI + ionization mode was performed. This enabled to carry out a qualitative and quantitative analysis of metabolite changes in GBEs. The liquid chromatography is capable of separating peaks detected by photodiode array detector (PDA) and then the mass spectrometer ionizes the compound to generate charged molecule and molecule fragment, measuring their mass-to-charge ratio.

**Figure 4:**
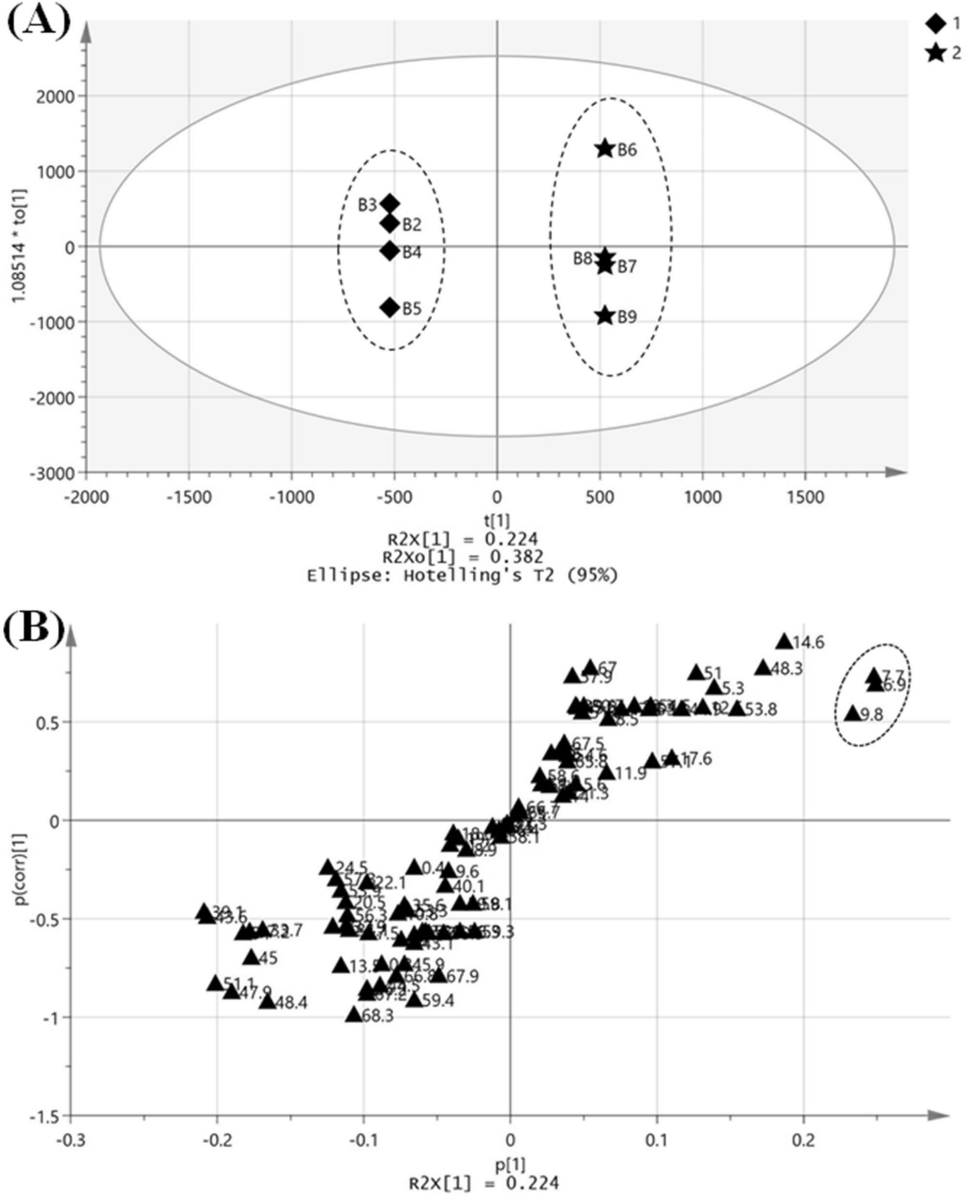
Orthogonal partial least-squares to latent structures-discrimination analysis (OPLS-DA) of characterizing components related to anti-wrinkle activity. (**A**) Score plot of OPLS-DA from UPLC–PDA chromatogram data of DW-GBEs and MRW-GBEs (1. Diamond represents DW-GBEs; 2. The 5-pointed star represents MRW-GBEs). (**B**) S-plot of OPLS-DA from UPLC–PDA chromatogram data of DW-GBEs and MRW-GBEs (Markers in the circle were the selected differential compounds of which the VIP > 2). All the plots were drawn by SIMCA-P + (Version 14.1).

In order to find out the characterizing compounds related to anti-wrinkle activity, the OPLS-DA was performed between DW-GBEs (B2, B3, B4, B5) and MRW-GBEs (B6, B7, B8, B9) by using the data of retention time and peak area. As shown in Fig. 4A, a clear separation between two groups was achieved. The R^2^X, R^2^Y, and Q^2^ were 0.959, 1 and 0.852 respectively, indicating the goodness of fit and excellent prediction ability of the model. In order to validate the reliabilities of OPLS-DA model, the permutation test was applied (n = 200). From the Score plot of OPLS-DA, three markers circled in Fig. 4B with significant covariance (the contribution of variations to the model, as X-axis) and correlation values (the reliability of variations to the model, as Y-axis) were considered to be the potential markers discriminating between two groups. The VIP values of these three markers were larger than 2, which reflected the importance of variables on the classification. Thus, the three markers were considered to be the characterizing compounds between DW-GBEs (B2, B3, B4, B5) and MRW-GBEs (B6, B7, B8, B9). It is estimated that the differences within the group B6 to B9 were caused by quantitative differences of active components and the differences within the group were not significant compare to the inter-group differences (Fig. 4A).

**Figure 5:**
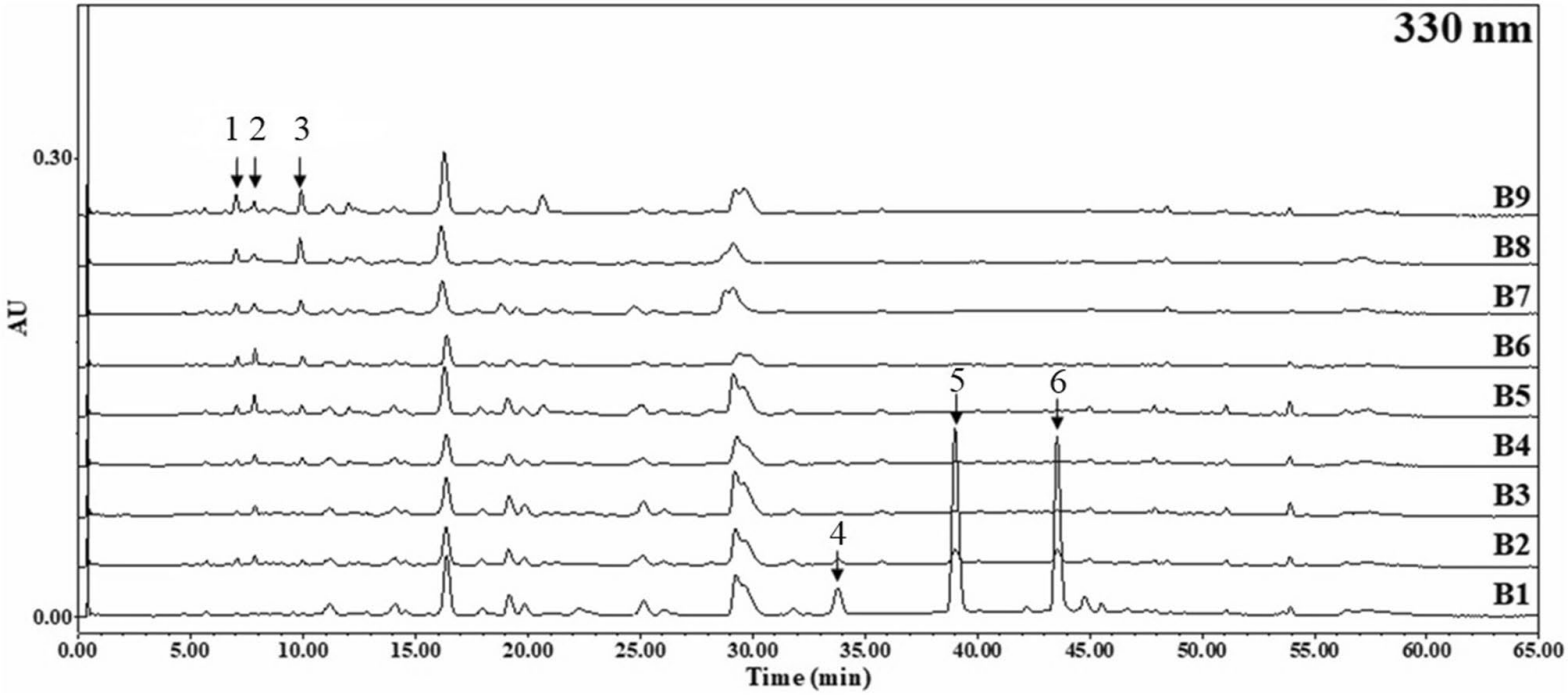
UPLC chromatogram at 330 nm for the comparison among samples of no germination (blank control; B1), DW-GBEs (B2, B3, B4, B5), and MRW-GBEs (B6, B7, B8, B9). The numbers of the peaks in this figure correspond to the compound numbers in Table 1.

The UPLC chromatograms (detected at 330 nm) of samples without germination (B1), DW-GBEs (B2, B3, B4, B5) and MRW-GBEs (B6, B7, B8, B9) were compared together and shown in Fig. 5. According to the retention time, the three marker compounds related to anti-wrinkle activity were numbered as 1 to 3. As shown in Fig. 5, these three peaks’ area has increased more in MRW-GBEs than DW-GBEs. Besides, there is a significant reduction of three peaks’ area of GBEs (B2 to B9) comparing with B1 that only soaked in DW for 3 h without germination. These three peaks were also marked in Fig. 5 and numbered as 4 to 6.

The mass spectra of the individual marker compounds were analyzed by comparison with previously published libraries and fragmentation profile. Peaks were also authenticated from masses and ultraviolet absorption maxima. With comprehensive analysis, peak 1, peak 2, and peak 3 were identified as the tetramer compounds of procyanidin (PC) and prodelphinidin (PD), which combined differently as PC1:PD3 and PC2:PD2^25^. Peak 2 and peak 3 were assumed to have same composition of PC and PD but different connecting location between them. PC and PD are members of the proanthocyanidin class of flavonoids. They are oligomeric compounds composed of flavan-3-ols molecules such as catechin, epicatechin, gallocatechin, and epigallocatechin. The polyhydroxy structure of PC and PD gives it special antioxidant activity and ability to scavenge free radicals^26–28^. Besides that, PC and PD have unique effects on skin aging caused by various factors, especially the anti-wrinkle effects based on its ability to maintain the synthesis of collagen and inhibit elastase. It has been reported that PC and PD help to synthesis collagen by protecting and regenerating Vitamin C because it can convert lysine and proline to hydroxylysine and hydroxyproline which is the main amino acids in collagen synthesis^29^. Peak 4, peak 5, and peak 6 were identified to be rosmarinic acid, luteolin and apigenin^25^, which have been previously identified in barley^30,31^. The information of six peaks was listed in Table 1.

**Table 1:** Retention time (R_t_), compound name, experimental mass number [M + H]^+^, and ultraviolet absorption maxima (λ_max_) of peaks.

	Rt (min)	Compound name	[M + H]^+^	λ max
1	6.9	PC1:PD3 tetramer^a^	1203.2588	218, 280
2	7.7	PC2:PD2 tetramer^a^	1187.2728	226, 306
3	9.8	PC2:PD2 tetramer^a^	1187.2701	224, 298
4	33.7	Rosmarinic acid	361.0920	218, 329
5	38.9	Luteolin	287.0516	253, 348
6	43.5	Apigenin	271.0572	266, 338

^a^PC and PD are abbreviations for procyanidin and prodelphinidin.

In conclusion, MRW-GBEs represented more potent anti-wrinkle activity on HDFs than DW-GBEs. The bioactive compounds related to the anti-wrinkle activity were specified to be oligomeric compounds of PC and PD using OPLS-DA study. We proposed that oligomeric compounds of PC and PD are the representative anti-wrinkle compounds significantly present in MRW-GBEs which may be a useful candidate ingredient for anti-wrinkle products. This study also provides a metabolomic template for further investigation into bioactive components of germinated cereal plants and its potential applications in cosmeceutical and pharmaceuticals.

## Supplementary Information

Supplementary Information.
