# Supplementary material for: Secondary metabolites changes in germinated barley and its relationship to anti-wrinkle activity
Source: Sci Rep. 2021 Jan 12;11:758. doi: 10.1038/s41598-020-80322-0 (PMC7804254; doi:10.1038/s41598-020-80322-0)
Supplement: Supplementary file 1 — Supplementary Information. [file 41598_2020_80322_MOESM1_ESM.docx]

**Secondary metabolites changes in germinated barley and its relationship to anti-wrinkle activity**

Sang Cheol Park^1^, Qianwen Wu^1^, Eun-yi Ko^2^, Ji Hwoon Baek^3^, Jeoungjin Ryu^4^, Seunghyun Kang^4^, Mi Kyung Sung^5^, Ah-Reum Cho^6^, Young Pyo Jang^1,7*^

^1^ Department of Life and Nanopharmaceutical Sciences, Kyung Hee University, Seoul, 02447, Republic of Korea

^2^ Dermapro Bio Research Center, DERMAPRO Ltd., 213-3 Chumdan-ro, Jeju-si, Jeju-do, 63309, Korea

^3^ Dermapro Skin Research Center, DERMAPRO Ltd., 30 Bangbaejoongang-ro, Seocho-gu, Seoul, 06684, Korea

^4^ Cosmax BTI R&I Center, Bio Material Research Team, 13486 Seongnam-si, Republic of Korea

^5^ Durae Corporation, Durae R&D Center, 15847 Gunpo-si, Republic of Korea

^6^ Durae Corporation, Jeju Bio Center, 63359 Jeju-si, Republic of Korea

^7^ Department of Oriental Pharmaceutical Science, Kyung Hee University, Seoul, 02447, Republic of Korea

*Corresponding author: Young Pyo Jang

Email addresses: [ypjang@khu.ac.kr](mailto:ypjang@khu.ac.kr)

Address: Department of Oriental Pharmaceutical Science, Kyung Hee University, Kyungheedae-ro 26, Dongdaemun-gu, Seoul, 02447, Republic of Korea

Tel: 82-2-961-9421

Fax: 82-2-961-9580


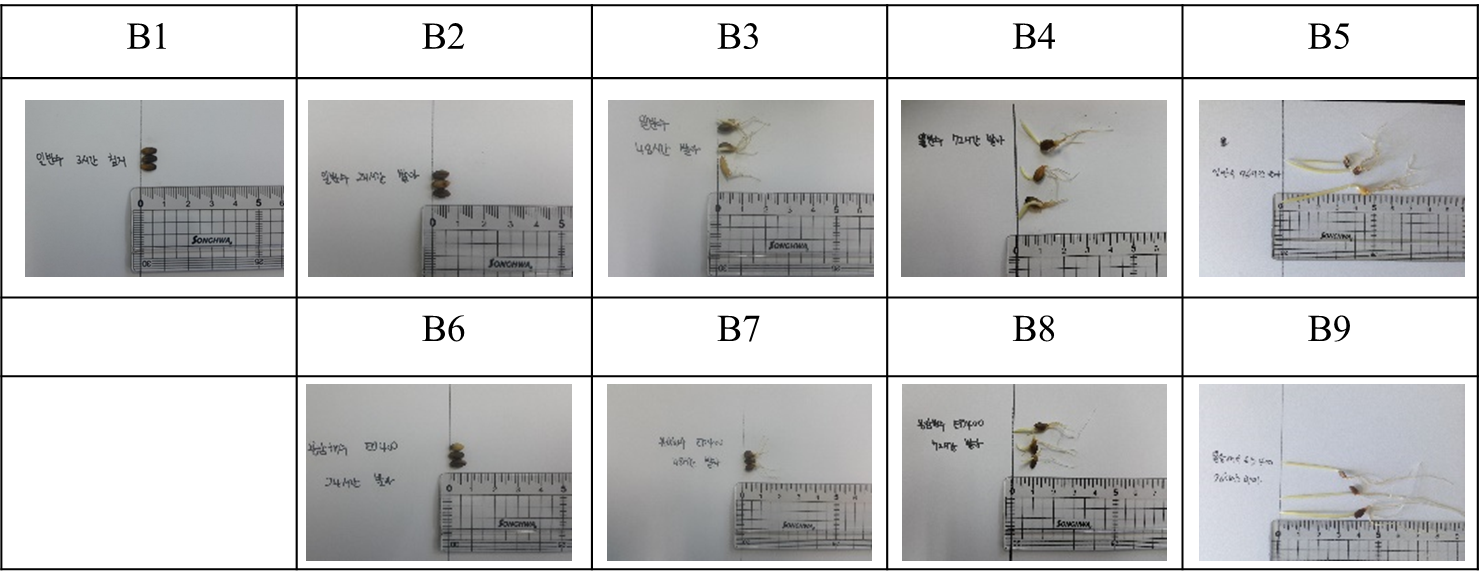


S. Figure 1. Photographs of germinated barley samples according to germination periods. B1: no germination, B2(DW), B6(MRW): 24-hr germination, B3(DW), B7(MRW): 48-hr germination, B4(DW), B8(MRW): 72-hr germination, B5(DW), B9(MRW): 96-hr germination.

S. Table 1. Root and stem length measurements of germinated barley samples

| Sample name | | B1 | B2 | B3 | B4 | B5 | B6 | B7 | B8 | B9 |
| --- | --- | --- | --- | --- | --- | --- | --- | --- | --- | --- |
| Length (mm) | Root | 0 | 0 | 13 | 16 | 33 | 0 | 13 | 22 | 60 |
|  | Stem | 0 | 0 | 7 | 12 | 40 | 0 | 0 | 10 | 40 |
